# Benefits of small-quantity lipid-based nutrient supplements for child nutrition and survival warrant moving to scale

**DOI:** 10.1038/s43016-023-00703-2

**Published:** 2023-02

**Authors:** Victor M. Aguayo, Shawn K. Baker, Kathryn G. Dewey, Emanuela Galasso, Abigail Perry, Zeina Sifri, Dina Aburmishan, Saskia de Pee, Saul I. Guerrero Oteyza, Grainne Moloney, Elizabeth L. Prado, Rahul Rawat, Linda Shaker-Berbari, Meera Shekar, Christine P. Stewart, Alison Tumilowicz, K. Ryan Wessells

**Affiliations:** 1 Nutrition and Child Development, Programme Division, UNICEF, New York, NY, USA; 2 US Agency for International Development (USAID), Washington, DC, USA; 3 Institute for Global Nutrition, Department of Nutrition, University of California, Davis, Davis, CA, USA; 4 Development Research Group, The World Bank Group, Washington, DC, USA; 5 Nutrition Division, United Nations World Food Programme, Rome, Italy; 6 Maternal, Newborn, and Child Health, Bill & Melinda Gates Foundation, Seattle, WA, USA; 7 Bureau of Humanitarian Assistance, US Agency for International Development (USAID), Washington, DC, USA; 8 Health, Nutrition and Population Global Practice, The World Bank Group, Washington, DC, USA; 9 Present address: Helen Keller Intl, New York, NY, USA

Better nutrition is critical for protecting children from disease and death in the short term, and for building human capital and life opportunities in the long term. Scaling up effective actions to improve child nutrition is more urgent than ever given that the current food and nutrition crisis is linked to rising child undernutrition, which drives 45% of deaths among young children^1^. Although recent commitments to increase funding for the early detection and treatment of child wasting are encouraging, we argue that prevention of wasting and other forms of child undernutrition using proven interventions should also be prioritized.

A relatively new and highly impactful preventive intervention was recently added to the list of approaches recommended to reduce child undernutrition: small-quantity lipid-based nutrient supplements (SQ-LNS) used to enrich the diets of children 6–23 months of age^2^. SQ-LNS were developed based on the same type of lipid-based food matrix used for ready-to-use therapeutic food (RUTF), which revolutionized strategies for the treatment of severe wasting; RUTF does not require preparation or refrigeration and thus can be administered in community settings for home-based treatment^3^. The usual daily dose of RUTF is large, typically more than 500 kcal per day, because it is intended for the treatment of young children who are severely wasted. SQ-LNS have a much smaller daily dose, typically less than 120 kcal per day (approximately 4 teaspoons). The goal is prevention of undernutrition, which requires a supplement that complements the daily diet rather than a food that constitutes a therapeutic diet to treat severe wasting. When developing SQ-LNS, the dose was intentionally kept as small as possible in order to avoid displacement of breastmilk and locally available nutrient-rich foods, to ensure that all children in the target age range can consume the entire ration in one day (thereby receiving the intended doses of the micronutrients) and to minimize cost^3^. SQ-LNS is a generic term that encompasses formulations available from various producers that use varying global and country-specific brand names; they are not yet available on the retail market but are listed in the Supply Catalogue of UNICEF, the United Nations Agency for Children^4^. The food base usually includes a vegetable oil rich in omega-3 fatty acids, a legume (such as peanut, chickpea, lentil or soybean) and milk powder, and the typical formulation is fortified with 22 vitamins and minerals, generally at levels approximating the daily recommended intake of each of these (micro)nutrients^5^. By including these nutrients along with the energy, high-quality protein and essential fatty acids provided by the food base, SQ-LNS addresses multiple potential nutritional deficiencies.

The evidence base for efficacy of SQ-LNS, based on meta-analyses of numerous randomized trials that included a total of more than 37,000 children, indicates reduced relative risk of multiple adverse outcomes including mortality (by 27%), severe wasting (by 31%), severe stunting (by 17%), iron deficiency anaemia (by 64%), and developmental delay (by 16–19%) between 6 and 23 months of age, as summarized in Table 1. Four studies of costs and cost-effectiveness suggest that SQ-LNS are a highly cost-effective intervention^6^. Our view is that SQ-LNS should be added to the ‘tool-kit’ of essential nutrition interventions promoting child survival, growth and development. SQ-LNS are not a stand-alone intervention, but when integrated into a core package of actions they can be protective in contexts where access to diverse diets is limited. At a minimum, the core package should include robust communication, counselling and support for continued breastfeeding and a diverse nutritious diet. Evidence from several SQ-LNS trials included in the meta-analyses indicates that adding SQ-LNS to high-quality communication and counselling interventions confers benefits that exceed those of the communication and counselling interventions alone^7^. In general, in populations in which child undernutrition is prevalent and child diets are severely poor, communication and counselling interventions are effective for improving infant and young-child feeding practices, but there is less evidence of effects on survival, growth, development, anaemia or micronutrient status^8^. Caregiving interventions that promote responsive care and early learning opportunities have larger effects on cognitive, language, motor and social–emotional development^9^ compared with SQ-LNS. However, these interventions do not seem to improve linear growth^7^ and effects on mortality or anaemia have not been demonstrated. Other fortified products such as micronutrient powders have effects on iron deficiency and anaemia that are similar to those of SQ-LNS^10^, but beneficial effects of micronutrient powders on survival, growth and development have not been reported in meta-analyses. To our knowledge, SQ-LNS are the only preventive intervention for children for which a simultaneous beneficial impact on all of these outcomes has been demonstrated in vulnerable populations, as described above. Based on this evidence, there is a growing consensus that SQ-LNS should be scaled up for children aged 6–23 months in need.

In 2021, a group of technical experts formed an informal planning committee to begin discussing how to achieve this goal, with members from several key organizations including UNICEF; the World Food Programme; the World Bank Group; the US Agency for International Development; the University of California, Davis; and the Bill & Melinda Gates Foundation. The first step was to organize a small convening in May 2022, supported by the Bill & Melinda Gates Foundation, including stakeholders representing donors, United Nations agencies, implementers, government partners and researchers. The goal was to create a shared vision for scaling up SQ-LNS within coordinated strategies to improve child survival, prevent child malnutrition and promote healthy growth and development. The convening focused in particular on operational issues for scale up, with the objective of creating a road map to prioritize next steps for the next 1–2 years.

Participants agreed that because SQ-LNS benefit numerous outcomes, they can be positioned as an evidence-based intervention within multiple relevant frameworks (for example, Child Survival Call to Action, Global Action Plan on Child Wasting, Human Capital Index and Nurturing Care Framework) and integrated within different programme delivery platforms. Provision of SQ-LNS should always occur alongside interventions to improve children’s diets, and be integrated into other existing interventions. Positioning good-quality complementary feeding enhanced with SQ-LNS within overall country strategies for improving child survival, nutrition and development is critical. In each country considering implementation, the vision of scale should include definitions of priority populations, delivery platforms, operational issues in various contexts, long-term plans for capacities and resources, opportunities for learning and course corrections, and clear indicators of programme monitoring and impact. Implementers should build on the lessons and best practices generated from programmes that have utilized SQ-LNS and similar products^5^.

Evidence shows that SQ-LNS can save lives, prevent malnutrition and enable children in need to grow and thrive. We believe that this intervention should be scaled up and integrated into programmes to promote maternal and child nutrition and well-being during the critical first 1,000 days in populations in which child undernutrition is prevalent and dietary quality is very poor. Collectively, we are committed to expanding access to SQ-LNS where they are needed most, supporting national governments to implement this intervention, identifying financing opportunities, evaluating programme impact particularly in settings with high levels of food insecurity, and promoting continued operational learning to optimize effectiveness.

## Figures and Tables

**Table 1 |:** Summary of relative reductions in adverse outcomes in SQ-LNS interventions

	Relative reduction % (95% CI)
**Growth** ^11,12^	
Stunting (LAZ <-2 SD)	12 (9, 15)
Severe stunting (LAZ <−3 SD)	17 (10, 22)
Wasting (WLZ <−2 SD) (cross-sectional prevalence)	14 (7, 20)
Severe wasting (WLZ <−3 SD) (cross-sectional prevalence)	31 (14, 45)
Underweight (WAZ <−2 SD)	13 (9, 17)
Acute malnutrition (WLZ <−2 SD or MUAC <125 mm)	14 (7, 20)
Low MUAC (MUACZ <−2 SD or MUAC <125 mm)	18 (11, 25)
Small head circumference (HCZ <−2 SD)	9 (5, 14)
**Development** ^13^	
Low language development score	16 (8, 24)
Low motor development score	16 (8, 24)
Low social–emotional development score	19 (11, 26)
**Anaemia and micronutrient status** ^14^	
Anaemia (Hb <110 g l^−1^)	16 (13, 19)
Moderate-severe anaemia (Hb <100 g l^−1^)	28 (24, 32)
Iron deficiency (ferritin <12 μg l^−1^)	56 (50, 61)
Iron deficiency anaemia (Hb <110 g l^−1^ and ferritin <12 μg l^−1^)	64 (56, 70)
Vitamin A deficiency (RBP <0.70 μmol l^−1^)	56 (30, 73)
**Mortality** ^15^	27 (11, 41)

Data are the relative reduction (95% confidence interval (CI)) in the prevalence of each outcome in the SQ-LNS group compared with the control group (which received no intervention other than standard messages promoting recommended feeding practices, or an intervention without any type of LNS or other child nutrition supplement) from meta-analyses of data from 14–18 randomized controlled trials. Some trials combined provision of child SQ-LNS with provision of maternal LNS, or with non-nutritional interventions (for example, improved water, sanitation and hygiene) in certain trial arms; in sensitivity analyses that excluded the maternal supplementation arms or isolated the comparisons to pairs of arms with the same non-nutrition components, results were very similar. Low development scores are defined as the lowest decile for each trial, based on the within-study distribution. Hb, haemoglobin concentration; HCZ, head circumference-for-age z-score; LAZ, length-for-age z-score; MUAC, mid-upper-arm circumference; MUACZ, mid-upper-arm-circumference z-score; RBP, retinol binding protein; SD, standard deviation; WAZ, weight-for-age z-score; WLZ, weight-for-length z-score.
